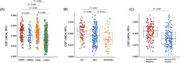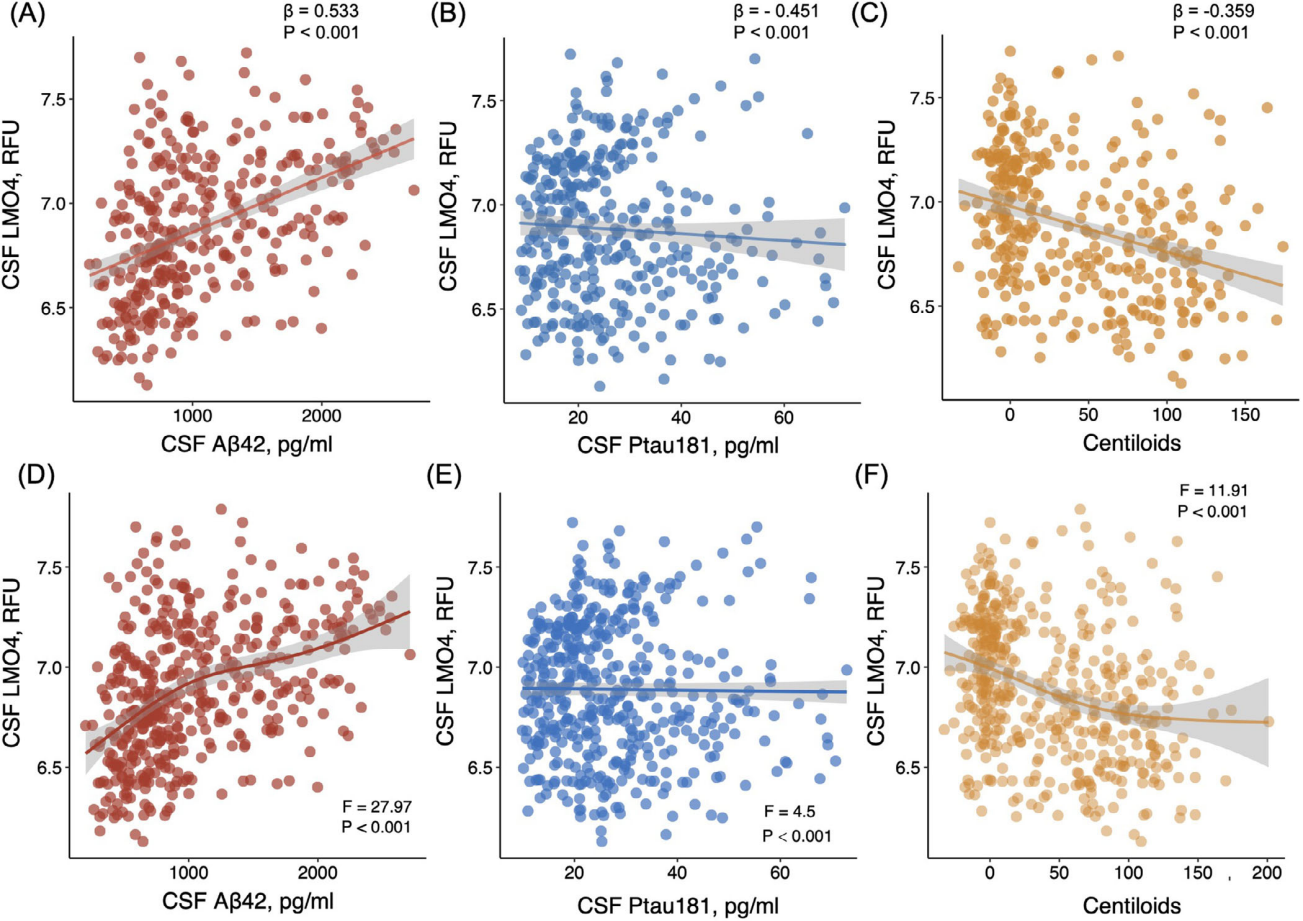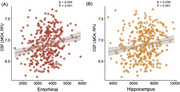# Cerebrospinal fluid LMO4 as a synaptic biomarker linked to Alzheimer's disease pathology and cognitive decline

**DOI:** 10.1002/alz70856_101808

**Published:** 2025-12-25

**Authors:** Yuhan Chen, Zhiqi Mao

**Affiliations:** ^1^ Hebei North University, Zhangjiakou, Hebei, China; ^2^ Chinese PLA General Hospital, Beijing, Beijing, China

## Abstract

**Background:**

LIM‐domain‐only 4 (LMO4) is involved in neurodevelopment and synaptic plasticity, but its role in the pathogenesis of Alzheimer's disease (AD) remains unclear.

**Method:**

We included 703 participants from the Alzheimer's Disease Neuroimaging Initiative (ADNI). Associations between CSF LMO4 and AD biomarkers (Aβ42, Ptau181, amyloid PET) and postmortem neuropathology were evaluated. Cross‐sectional and longitudinal associations between CSF LMO4 and neurodegeneration and cognitive function were explored. Receiver operating characteristic analysis assessed the diagnostic accuracy of CSF LMO4 in distinguishing Aβ‐positive from Aβ‐negative participants and amyloid PET‐confirmed AD cases. Mediation analysis explored the potential mediating role of CSF LMO4 between Aβ pathology and tau pathology.

**Result:**

LMO4 levels were decreased in participants with abnormal Aβ levels and cognitive impairment. Lower CSF LMO4 levels were associated with increased Aβ and tau pathology, brain atrophy, cognitive decline, and postmortem neuropathology. CSF LMO4 partially mediated the relationship between Aβ and tau pathology and demonstrated acceptable discriminative ability in distinguishing Aβ‐positive from Aβ‐negative participants and amyloid PET‐confirmed AD from non‐AD cases.

**Conclusion:**

CSF LMO4 plays a crucial role in the pathogenesis and progression of AD and may represent a potential therapeutic target for AD treatment.